# Clinical values of multiple **Epstein-Barr virus **(EBV) serological biomarkers detected by xMAP technology

**DOI:** 10.1186/1479-5876-7-73

**Published:** 2009-08-23

**Authors:** Ai-Di Gu, Li-Xia Lu, Yan-Bo Xie, Li-Zhen Chen, Qi-Sheng Feng, Tiebang Kang, Wei-Hua Jia, Yi-Xin Zeng

**Affiliations:** 1 State Key Laboratory of Oncology in Southern China, Guangzhou, PR China; 2 Department of Experimental Research, Sun Yat-sen University Cancer Center, Guangzhou, PR China; 3 Department of Radiotherapy, Sun Yat-sen University Cancer Center, Guangzhou, PR China

## Abstract

**Background:**

Serological examination of Epstein-Barr virus (EBV) antibodies has been performed for screening nasopharyngeal carcinoma (NPC) and other EBV-associated diseases.

**Methods:**

By using xMAP technology, we examined immunoglobulin (Ig) A antibodies against Epstein-Barr virus (EBV) VCA-gp125, p18 and IgA/IgG against EA-D, EBNA1 and gp78 in populations with distinct diseases, or with different genetic or geographic background. Sera from Cantonese NPC patients (n = 547) and healthy controls (n = 542), 90 members of high-risk NPC families and 52 non-endemic healthy individuals were tested. Thirty-five of NPC patients were recruited to observe the kinetics of EBV antibody levels during and after treatment. Patients with other EBV-associated diseases were collected, including 16 with infectious mononucleosis, 28 with nasal NK/T cell lymphoma and 14 with Hodgkin's disease.

**Results:**

Both the sensitivity and specificity of each marker for NPC diagnosis ranged 61–84%, but if combined, they could reach to 84.5% and 92.4%, respectively. Almost half of NPC patients displayed decreased EBV immunoactivities shortly after therapy and tumor recurrence was accompanied with high EBV antibody reactivates. Neither the unaffected members from high-risk NPC families nor non-endemic healthy population showed statistically different EBV antibody levels compared with endemic controls. Moreover, elevated levels of specific antibodies were observed in other EBV-associated diseases, but all were lower than those in NPC.

**Conclusion:**

Combined EBV serological biomarkers could improve the diagnostic values for NPC. Diverse EBV serological spectrums presented in populations with different EBV-associated diseases, but NPC patients have the highest EBV activity.

## Background

Epstein-Barr virus (EBV) is a ubiquitous γ-herpesvirus which infects more than 90% of the worldwide population [1]. In developing countries, primary EBV infection usually occurs in the childhood and is asymptomatic [2]. But in western countries, primary infection with EBV can be delayed until adolescence with occurrence of infectious mononucleosis (IM) [3]. EBV could establish a life-long persistent infection without serious consequences in most of populations, but a number of documents showed that EBV infection was involved in many diseases, including Hodgkin's disease (HD) [4], gastric cancer and lymphoproliferative diseases [5,6]. Interestingly, EBV is also associated with some specific cancers with endemic patterns [7], such as nasopharyngeal carcinoma (NPC) in south China and Southeast Asia [8], Burkitt's lymphoma (BL) in equatorial Africa and Papua New Guinea [9], nasal NK/T-cell lymphoma in Asia and Latin American [10].

Generally, people infected by EBV will develop specific antibodies against this virus, even with primary infection including IM, which is characterized by the first presence of immunoglobulin (Ig) M antibodies against viral capsid antigen (VCA) and followed by IgG against VCA, early antigen (EA) and EBV nuclear antigen 1 (EBNA1) [11]. On the other hand, aberrant antibody levels against EBV have been evidenced in the EBV-associated carcinomas due to the specific EBV gene-expression patterns [8]. For instance, anti-VCA and anti-EA antibody levels are increased in BL and HD patients prior to and/or at the time of diagnosis [12]. NPC patients usually have high IgA and/or IgG reactivities to various EBV antigens, including VCA, EA, EBNA1, transcription activator Zta and Rta, etc [13-16]. Notably, the elevated EBV antibody responses could precede the clinical onset of NPC by 1–5 years [17,18], indicating that the examination of EBV antibodies is valuable for the diagnosis NPC. In addition, prognosis of NPC could be reflected by the fluctuation of EBV antibody levels after NPC therapy [19]. Thus, EBV serological examination may be crucial for the diagnosis and prognosis of NPC.

Molecular diversity of EBV serological profiles in NPC patients has been visualized by immunoblot method and thereby simultaneous examination of several EBV biomarkers could improve the efficiency of NPC diagnosis [20]. At present, Luminex multi-analyte profiling (xMAP) technology has been developed, in which more than one hundred distinct reactions could be carried out simultaneously [21]. Based on this technology, we have recently reported that IgA- and IgG-gp78 are novel biomarkers for NPC diagnosis by screening EBV serological parameters [22]. In this study, we performed EBV serological examination with 8 EBV biomarkers in a large scale of Cantonese NPC patients and healthy controls in order to value their clinical values. In addition, various EBV serological profiles were also revealed among different populations, such as the high-risk NPC families, the non-endemic healthy controls and patients with other EBV-associated diseases.

## Methods and Materials

### Study populations

A total of 547 NPC patients and 542 healthy controls from Cantonese population were included in this study. These NPC patients were newly diagnosed and pathologically confirmed. The stage of disease progression was classified according to the 1996 Union International Cancer Control classification. The NPC case group included 17 at cancer stage I, 90 at stage II, 286 at stage III and 154 at stage IV. The healthy volunteers were collected as controls (Table 1). Additional 35 NPC patients were recruited to study their EBV antibody levels before, during and after treatment. The patients were followed-up for 3–12 months. Moreover, 92 individuals were derived from 6 high-risk NPC families, with at least two NPC cases in each family. 52 sera from the low-risk healthy controls were collected in Shanxi Province, a non-endemic NPC area in north China.

**Table 1:** Characteristics of Cantonese healthy controls and NPC patients

	Healthy (n)		NPC (n)		NPC cancer stage (n)			
			
Age rang (years)	male	female	male	female	stage I	stage II	stage III	stage IV
< 30	29	38	13	9	2	5	10	5
30–39	73	52	89	37	0	23	73	30
40–49	78	61	127	50	10	31	85	51
50–59	86	46	109	38	4	24	79	40
≧ 60	58	21	53	22	1	7	39	28

**total**	324	218	391	156	17	90	286	154

**NOTE**. Data are sample volumes in this study.

Sera from patients with other diseases were obtained from the serum repository at Sun Yat-Sen University Cancer Center. Children with IM (n = 16), suffering from fever, pharyngitis and lymphodenopathy, were diagnosed by the presence of anti-VCA IgM. Patients with HD (n = 14), nasal NK/T cell lymphoma (n = 28), and other non-Hodgkin's lymphoma (NHL) (n = 49) were confirmed by histopathology. Patients with non-NPC solid tumors were collected, including head and neck tumours (n = 94), lung cancer (n = 49), stomach cancer (n = 19) and intestinal cancer (n = 27). The Institutional Review Board of our hospital approved this study and written informed consents were obtained from these participants.

### xMAP technology

#### Synthetic peptide

Immunodominant epitopes on VCA-p18, EBNA1 and gp78 were defined as described before [23]. Briefly, the protein sequences were examined by DNAStar software according to the reported EBV proteomes [24]. The sequences with high possibility of hydrophilicity, surface-orientation and flexibility were chosen. About 20 residues of each peptide were selected and synthesized by adding six carbon and one biotin at the N terminus (Hanyu, China), and then further purified by high-performance liquid chromatography to achieve > 90% purity. The peptide sequences used in this study were shown as follows: p18 (BFRF3), GGGQPHDTAPRGARKKQ; EBNA1 (BKRF1), GSGPRHRDGVRRPQKRPS; gp78 (BILF2), TSTSHRPHRRPVSKRPTHK.

### xMAP analysis

Coupling of recombinant EBVVCA-gp125, EA-D (Biodesign) to the carboxylated beads (Luminex) and biotinylated peptides to LumAvidin microspheres (Luminex) was performed according to the manufacturer's instruction. Details and interpretation of the procedure have been described before [22,25].

The conjugated beads were diluted with storage buffer according to 1000 beads/50 μl per reaction well and then added to the 96-well filtration system (Millipore). Sera diluted to 1:21 in storage buffer (20 μl/well) were added and incubated with the beads for 30 min and protected from light at room temperature. After washing thrice, 150 μl of R-phycoerythrin (R-PE) conjugated goat anti-human IgA or IgG (Jackson ImmunoResearch, 1:200 in PBS) was added to each reaction well and incubated for 30 min. The detection analysis was performed by Luminex multi-analytic system 100 (Bio-Rad). All tests were carried out in duplicate.

### Statistical Analysis

The results were analyzed using the statistics software SPSS (v. 16.0). The unpaired *t *test was used to compare the mean values from NPC and healthy groups. Receiver operating characteristic (ROC) curve analysis was done to determine the cutoff values. Logistic regression was used to create a diagnostic model of NPC. One-way analysis of variance (ANOVA) was used to compare mean fluorescence intensity (FI) of all EBV biomarkers among NPC patients with different ages and cancer stages or other patients with different diseases.

## Results

### Diagnostic values of multiple EBV biomarkers for NPC

By using in-house xMAP assays, we analyzed 8 EBV antibodies, including 5 IgA antibodies against VCA-gp125, p18, EA-D, EBNA1 or gp78 and 3 IgG antibodies to EA-D, EBNA1 or gp78, in a large scale of Cantonese healthy subjects and NPC patients. The mean FI values for each antibody were significantly higher in NPC patients than that in healthy controls (*P *< 0.05) (see Additional file 1). Therefore, ROC analysis was consequently utilized to check the diagnostic values of these serological biomarkers for NPC. As shown in Table 2, the areas under ROC curve (AUCs) of IgA-EBNA1, IgA-EA-D and IgG-EA-D were 0.81 (95% CI, 0.79–0.84), 0.87 (95% CI, 0.85–0.89) and 0.90 (95% CI, 0.88–0.92), respectively, whereas those of other biomarkers ranged from 0.68 to 0.77. In addition, based on the ROC analysis, the cutoff FI values were also determined. Interestingly, 52 of 542 (9.6%) healthy controls have lower FI values than the cutoffs for all eight EBV parameters, consistent with the fact that more than 90% people worldwide are infected by EBV. For all eight biomarkers, only 0.4% of NPC patients had false negative and 0.4% of healthy controls had false positive. Moreover, 92.6% of NPC patients had higher levels of at least four markers than the cutoff values, indicating that the eight parameters may be complementary for NPC diagnosis. Therefore, we performed logistic regression analysis to establish a diagnostic model for NPC using the 8 EBV parameters. In this model, the sensitivity and specificity were increased to 84.5% and 92.4%, respectively, much higher than single EBV biomarkers, further supporting our conclusion drawn recently [22].

**Table 2:** ROC analysis of EBV serological parameters for NPC diagnosis

EBV biomarker	AUC (95% CI)	Cutoff (FI)	Sensitivity (95% CI)	Specificity (95% CI)
IgA – EA-D	0.87 (0.85–0.89)	500	80% (77–83%)	77% (73–80%)
IgA – gp125	0.78 (0.75–0.80)	700	69% (65–73%)	73% (69–77%)
IgA – EBNA1	0.81 (0.79–0.84)	300	70% (66–74%)	76% (72–80%)
IgA – gp78	0.76 (0.73–0.79)	300	66% (62–70%)	72% (68–76%)
IgA – p18	0.72 (0.69–0.75)	500	60% (56–64%)	74% (70–78%)
IgG – EA-D	0.90 (0.88–0.92)	1000	81% (77–84%)	84% (81–87%)
IgG – EBNA1	0.68 (0.65–0.71)	1400	67% (63–71%)	61% (57–65%)
IgG – gp78	0.74 (0.71–0.76)	1600	62% (58–66%)	71% (67–74%)

**NOTE**. ROC analysis is made by using the data from the Cantonese panel, including sera from healthy subjects (n = 542) and NPC patients (n = 547) detected by xMAP technology. AUC, area under ROC curve; FI, fluorescence intensity; CI, confidence interval; EA-D, early antigen-diffused; EBNA1, Epstein-Barr nuclear antigen 1.

### EBV antibody levels in Cantonese subgroups with different characteristics

To assess the relationship between EBV antibody concentrations and cancer stages, ANOVA analysis was performed. Both IgA and IgG levels against EA-D increase gradually from lower NPC stages to higher NPC stages, and there are statistically different (*P *< 0.05) between any two NPC stages. For the stage II and stage IV NPC, there are also statistically different (*P *< 0.05) for IgA-VCA, IgA-gp78 and IgG-gp78 (data not shown). However, no statistic differences were observed between stage I and stage II or IV NPC. Collectively, our results suggest that later NPC stages have the tendency to induce more EBV antibodies.

Most of the EBV biomarker levels were independent of their ages for NPC patients. Unexpectedly, anti-EBV antibody levels increased in elder healthy populations. For example, sixties had higher levels than any of the other groups for IgA-p18 and IgG-gp78 and twenties had lower levels than any of the other groups for IgA-EA and IgA-gp78, both with statistic differences. In addition, there is no significant correlation between gender and any of the EBV biomarkers (data not shown).

### Kinetics of EBV antibody levels during NPC treatment

To examine the fluctuations of EBV antibody reactivities in NPC patients, we recruited 35 patients to perform a serial analysis of these EBV parameters during NPC treatment and follow-up. In the most patients, the kinetics of the eight anti-EBV antibodies was consistent.

**Figure 1:**
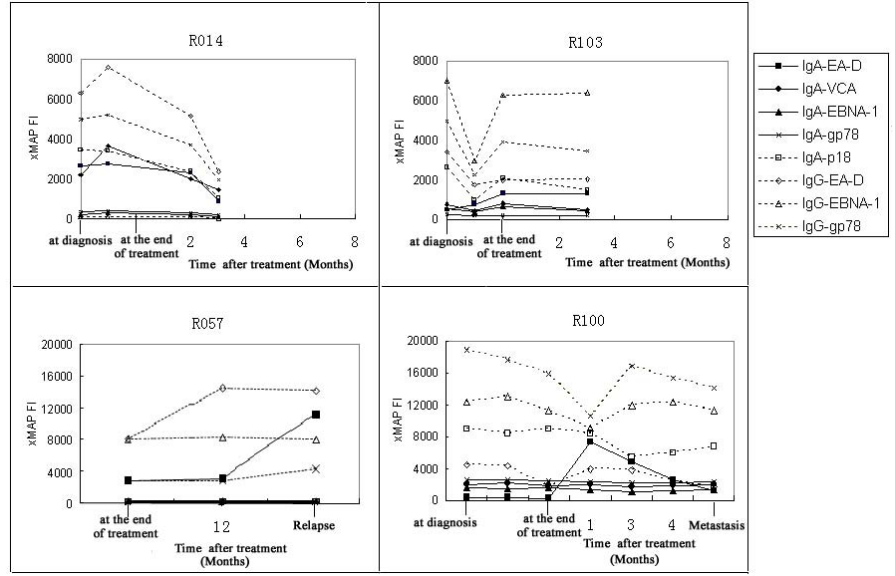
**Fluctuations of EBV antibody levels in four representative NPC patients during and after radiotherapy and/or chemotherapy**. The Y axis represents the mean xMAP fluorescence intensity (FI) for each EBV parameter. X axis, time from the start of blood sampling, with the day of diagnosis, the day of treatment end and the follow-up period. R014, R103, R57 and R100 represent different NPC patients.

In 15 of the 35 patients the levels of anti-EBV antibodies descended after the therapy while 13 showed small changes during the follow-up. However, the EBV antibody levels in 5 patients rose up after therapy and 2 patients firstly fell down and then rose up. For patient R014, the xMAP FI of IgG-EA-D was 6303 before treatment and then rose to 7567 after the initial chemotherapy, but it dropped to 2385 three months after therapy (Fig. 1). The initial rise of some EBV antibodies in patients R072, R077, R139 after clinical therapy all reached to ~40 – 70%. Interestingly, the reactivities of IgG-EBNA1 in patient R103 had a drastic decrease after the starting treatment, with xMAP FIs ranging from 7014 to 2970, whereas it ascend to 6279 at the end of treatment (Fig. 1). The disparity of EBV serological kinetics in different NPC individuals during treatment might reflect the different radiosensitivity and immunological reactivation.

Moreover, patient R057 showed continuous elevation of EBV immunoactivities one year after treatment. When NPC recurrence was detected, the antibody levels were much higher than those of pretreatment. But patient R100 showed a more complicated kinetics of EBV antibody reactivities. During the therapy, all of the EBV biomarkers fell down largely or slightly. However, the levels of IgA- and IgG-EA-D in patient R100 rose up at one month after finishing clinical treatment, whereas IgG-EBNA1 and IgG-gp78 had an elevation at three months. But IgA-p18 kept rising at four months and the time metastasis was detected (Fig. 1).

### EBV serological examination in the high-risk NPC families

In order to evaluate the distribution of EBV antibody levels in NPC high-risk families, we collected 92 sera from members of 6 families with at least two NPC patients for each family, including 15 NPC patients, 60 Grade I relatives and 17 Grade II relatives, based on their relationship to the NPC cases in the family: Grade I (parents, children, siblings) and Grade II (spouses).

Compared with the general NPC cases, the NPC individuals in the high-risk families showed lower EBV antibody levels except for IgA-EA (see Additional file 1). This may be due to the fact that a majority of the familiar cases in our study were after NPC therapy and the EBV seroreactivity declined. On the other hand, the unaffected individuals from high-risk families and community controls showed no statistical differences in the antibody levels against any EBV markers. Intriguingly, a couple with both NPC cases, who are from two separate high-risk families, didn't show elevated EBV antibody levels and their children are healthy.

### EBV serological detection in non-endemic healthy population and patients with other solid cancers

To compare the EBV antibody levels in populations from distinct geographic origins, we collected 52 sera of healthy blood donors from Shanxi Province, which is located in the north China and represents a non-endemic NPC area. The mean FI value in non-endemic healthy subjects were lower than those in Cantonese population for each EBV biomarker tested, although there was no statistical difference (see Additional file 1). Furthermore, we also examined sera from patients with other solid cancers. There was no difference for antibody levels of each EBV marker between any group of the patients and Cantonese controls.

### EBV serological profiles in patients with other EBV-associated diseases

We further analyzed these 8 EBV antibodies in sera from patients with different EBV-associated diseases. The mean FI values of theses markers are also presented in Additional file 1. Interestingly, all of these disease groups had much lower EBV antibody levels than NPC group. When compared with Cantonese healthy controls, IM patients had significantly higher IgA-gp125 level (*P *= 0.01) but relatively lower IgG levels. The IgA-p18 level in HD patients was higher than that in healthy group, but lower than that in NPC patients. However, neither was statistically different (*P *> 0.05). This may be due to a small number of HD patients. Compared with the healthy, patients with NK/T cell tumors had a significantly higher levels of IgG-EA (*P *= 0.03), and higher levels of IgA-EA and IgA-gp125 (*P *> 0.05), and a lower level of IgG-gp78 (*P *> 0.05); patients with NHL except for NK/T cell tumors had higher levels of IgA-EA and IgG-EBNA1 (*P *> 0.05). The results may indicate that EBV has different activities in various EBV-associated diseases.

## Discussion

EBV serology testing is usually performed by indirect immuno-fluorescence assay, ELISA or immunoblot [20,23,26], but these methods could only address one of two aspects: evaluation of EBV antibody parameters for the diagnosis of NPC or analysis of molecular diversity of EBV serological spectrums in different populations. In contrast, xMAP assay could achieve both simultaneously. At present, by using xMAP technology, we examined IgA and IgG levels against a wide spectrum of EBV antigens in populations with distinct diseases, or with different genetic or geographic background.

We are presenting a diagnostic model for NPC using logistic regression by combining 8 EBV biomarkers. This model could reach the sensitivity and specificity of 84.5% and 92.4%, respectively, to discriminate between NPC patients and healthy controls. Furthermore, this model could be further used to predict the risk rate of NPC occurrence in a large-scale screening. In addition, our study also confirmed that single EBV biomarker was not efficient enough for NPC diagnosis [20,23,27], and that there is a diversity of EBV-antigen-recognition spectrum within individuals [20].

Although EBV serological examination has been widely employed for assisting in NPC diagnosis, the temporal kinetics of antibody levels in a short period during and after treatment had been rarely studied. It was reported that patients with confirmed clinical recurrence 1 year after completion of radiotherapy had significantly increasing IgG-EA and mainly IgA-EA titers [28]. By using xMAP technology, we found the EBV antibody levels were also correlated with early clinical events of NPC patients after treatment, similar to the studies of plasma EBV DNA [29]. At the time of tumor recurrence, increased EBV antibody levels were observed. In some patients, an initial rise of EBV antibody reactivities was detected during NPC treatment, comparable to the initial rise of plasma EBV DNA after therapy [30]. So it strongly supports the close link of EBV antibody levels and NPC tumor load.

Familial history is one of the contributors to the risk of NPC [31-33]. EBV serology testing in Taiwan indicated that unaffected members of high-risk families had increased seropositivity rate of anti-VCA IgA and anti-EBNA1 IgA compared to general healthy population, but this trend was not observed among Greenlandic Inuit [34,35]. Our present study using the eight EBV markers showed that the percentage of positive subjects was identical in the healthy populations from either high-risk NPC family or community. The inconsistency might be due to the distinct age distributions among these studies, since elder healthy populations usually have higher anti-EBV antibody levels, which is another interesting finding in our study. Furthermore, our results showed that no statistical difference is observed between unaffected individuals of high-risk families and general controls for all EBV antibody levels tested, neither is between first-degree relatives and spouses of NPC cases. These are in agreement with previous studies [34,35]. But a long-term follow-up study on EBV antibody-elevated population from Taiwan suggested a significantly higher risk for developing into NPC than controls [17]. Therefore, EBV infection might not be the key initiator for NPC, but play an important role in the high-risk subjects. Other factors such as genetic susceptibility and environmental factors may be essential for the incidence and development of NPC as indicated previously [36-40].

EBV-associated diseases could be characterized by different EBV serological features. For example, the acute EBV infection resulted in IM could be reflected by the appearance of anti-VCA antibodies [41], which support our results showing that IgA-VCA gp125 levels significantly increased in IM patients as previously reported [42]. On the other hand, chronic EBV infection is linked to several lymphoma diseases with aberrant EBV antibody levels. HD patients usually have elevated IgG antibodies against VCA, EA-D and EBNA1 [43]. Interestingly, we found that compared with healthy controls, IgA-p18 was higher in HD patients and IgG-EA-D was higher in the patients with nasal NK/T cell lymphoma. Remarkably, NPC patients have higher levels of both IgA and IgG classes in a large spectrum of EBV antigens including VCA gp125 and p18, EA-D, EBNA1 and gp78 compared with the healthy populations or populations with other EBV-associated diseases, indicative of a vigorous viral activity in NPC.

Although viral expression in most EBV-associated tumor cells is mainly latent, transcription of a variety of lytic genes was detected in infiltrating lymphoid cells in NPC by in situ studies [44,45]. Accordingly, it has been suggested that diverse EBV antigens in the lymphoid stroma of NPC could stimulate EBV antibody reactivity and contributed to the specific serologic feature of this disease [46]. But the mechanism of uncontrolled EBV activity in cancer patients remains unclear. Depressed immune control of the virus might enable EBV more activated, since increasing EBV antibody levels were usually found in advanced cancer stage and aging healthy people, which have lower immunity [47]. In agreement with this speculation, reactivation of latent EBV infection was considered an important pathogenic mechanism of EBV-related diseases in immunocompromised patients such as those with PTLD or HIV [48,49]. However, patients with other solid tumors didn't show higher EBV activities than healthy controls in this study, suggesting EBV propagation may undergo in parallel with strong microenvironment disposition. Further investigations are awaited to characterize the biological activities and functions of EBV in NPC and lymphoma.

## Conclusion

Our results revealed that diverse EBV antibody spectrums presented in distinct populations with different EBV-associated diseases. Moreover, NPC individuals have various EBV serological profiles and combined EBV biomarkers could improve the analytic accuracy for diagnosis.

## Competing interests

The authors declare that they have no competing interests.

## Authors' contributions

YXZ and YBX were responsible for the design of this study. ADG carried out the experiments and drafted the manuscript. LXL participated in the data analysis. LZC, QSF and WHJ helped in serum samples colletion. TK helped in amending the manuscript. All authors read and approved the final manuscript.

## Supplementary Material

Additional file 1 Distribution of EBV serological biomarkers in different populations. Click here for file
