# Supplementary material for: Clinical values of multiple Epstein-Barr virus (EBV) serological biomarkers detected by xMAP technology
Source: J Transl Med. 2009 Aug 23;7:73. doi: 10.1186/1479-5876-7-73 (PMC2734757; doi:10.1186/1479-5876-7-73)
Supplement: Additional file 1 — Distribution of EBV serological biomarkers in different populations. [file 1479-5876-7-73-S1.doc]

**Additional file 1. Distribution of EBV serological biomarkers** in different populations.

| group |  | No. of sera | Mean antibody levels (MFI ±SEM) | | | | | | | |
| --- | --- | --- | --- | --- | --- | --- | --- | --- | --- | --- |
|  |  |  | IgA-EA | IgA-gp125 | IgA-EBNA1 | IgA-gp78 | IgA-p18 | IgG-EA | IgG-EBNA1 | IgG-gp78 |
| Cantonese controls ^a^ | | 542 | 402.6 ± 22.6 | 708.4 ± 35.1 | 403.6 ± 46.7 | 364.2 ± 21.8 | 1069.6 ± 107.2 | 936.6 ± 84.5 | 2635.1 ± 187.7 | 2178.4 ± 133.3 |
| Cantonese NPC | | 547 | 3836.6 ± 226.9^b^ | 2570.7 ± 131.7^b^ | 2302.6 ± 150.2 ^b^ | 1392.5 ± 111.6 ^b^ | 3277.1 ± 203.0^b^ | 7006.5 ± 251.6 ^b^ | 5744.3 ± 261.3 ^b^ | 6478.8 ± 240.8 ^b^ |
| high-risk NPC family members | |  |  |  |  |  |  |  |  |  |
|  | NPC patients | 15 | 3366.3 ± 1212.1 | 1838.3 ± 622.8 | 669.6 ± 193.7 | 799.6 ± 356.7 | 2165.7 ± 648.6 | 3557.3 ± 971.6 | 3644.9 ± 1278.5 | 2492.9 ± 914.3 |
|  | Grade I^c^ | 60 | 384.5 ± 38.3 | 717.9 ± 159.9 | 463.4 ± 203.8 | 201.4 ± 20.8 | 1171.3 ± 340.7 | 624.4 ± 133.5 | 1854.0 ± 370.7 | 1698.7 ± 316.4 |
|  | Grade II^d^ | 17 | 584.7 ± 101.7 | 826.9 ± 129.3 | 379.2 ± 91.2 | 352.2 ± 91.7 | 807.7 ± 240.5 | 951.6 ± 535.1 | 2992.6 ± 1077.6 | 2697.4 ± 593.4 |
| Non-endemic healthy | | 52 | 305 ± 26 | 428.8 ± 72.1 | 268.5 ± 68.4 | 313.8 ± 86.9 | 943.3 ± 430.4 | 638.5 ± 195.2 | 2430.3 ± 535.5 | 1188.4 ± 314.3 |
| Other solid tumors | |  |  |  |  |  |  |  |  |  |
|  | Head& neck tumors except for NPC | 94 | 650.7 ± 152.0 | 865.6 ± 193.7 | 529.9 ± 123.5 | 489.5 ± 101.5 | 1552.6 ± 249.7 | 917.9 ± 203.2 | 2100.1 ± 377.1 | 2772.7 ± 349.7 |
|  | Other tumors | 95 | 392.1 ± 48.5 | 536.2 ± 109.6 | 340.7 ± 43.9 | 432.5 ± 93.0 | 1006.3 ± 282.1 | 844.9 ± 265.4 | 2208.5 ± 382.7 | 1799.7 ± 274.7 |
| Other EBV-associated diseases | |  |  |  |  |  |  |  |  |  |
|  | IM | 16 | 704.2 ± 289.0 | 2034.8 ± 792.5 ^b^ | 215.6 ± 41.3 | 441.3 ± 100.9 | 536.5 ± 128.1 | 729.2 ± 262.1 | 1647.3 ± 806.2 | 1099.5 ± 302.7 |
|  | HD | 14 | 517.2 ± 136.9 | 892.5 ± 206.2 | 509.1 ± 185.8 | 566.1 ± 140.7 | 2318.0 ± 1207.1 | 803.8 ± 294.5 | 2621.9 ± 1357.7 | 2233.4 ± 967.6 |
|  | NK/T cell lymphoma | 28 | 789.1 ± 216.8 | 833.9 ± 202.8 | 357.2 ± 108.2 | 444.7 ± 81.4 | 1679.2 ± 511.8 | 2525.9 ± 821.1 ^b^ | 1425.2 ± 570.7 | 1581.3 ± 424.9 |
|  | other NHL | 49 | 781.7 ± 230.9 | 563.1 ± 111.6 | 272.2 ± 44.7 | 366.0 ± 44.5 | 743.1 ± 160.5 | 1081.2 ± 456.8 | 3363.5 ± 725.5 | 1588.6 ± 254.6 |

**NOTE**. Sera from populations with different disease conditions or ethnic backgrounds were detected for EBV serology using xMAP technology. Shown here are the FI values (mean ± standard error for mean (SEM)) of EBV biomarkers for each condition: Cantonese healthy controls, Cantonese patients with nasopharyngeal carcinoma (NPC), members from high-risk NPC families, non-endemic healthy subjects from Shanxi Province, solid tumors except for NPC and other EBV-associated diseases, including infectious mononucleosis (IM), Hodgkin’s disease (HD), nasal NK/T cell lymphoma and non-Hodgkin’s lymphoma except for NKT (NHL).

ANOVA analysis was performed to compare mean FI values of different populations with Cantonese healthy controls.

^a^ Reference

^b^ *P* <0.05

In the high-risk NPC families, unaffected members were classified according to their relationship to the NPC cases.

^c^ parents, children, siblings

^d^ spouses
